# Herpes simplex keratitis following Smart Pulse Technology assisted transepithelial photorefractive keratectomy: a case report

**DOI:** 10.1186/s12886-022-02654-x

**Published:** 2022-11-16

**Authors:** Ai-qin Nie, Xiao-min Chen, Qiang Li

**Affiliations:** Department of Ophthalmology, Shenzhen People’s Hospital (The Second Clinical Medical College, Jinan University; The First Affiliated Hospital, Southern University of Science and Technology), Shenzhen, 518020 Guangdong China

**Keywords:** Case report, Herpes simplex keratitis, SmartSurf^ACE^, Refractive surgery

## Abstract

**Background:**

Herpes simplex keratitis (HSK) is a rare and sight-threatening complication following refractive surgery. SmartSurf^ACE^ surgery is the result of combining transepithelial photorefractive keratectomy (trans-PRK) with Smart Pulse Technology (SPT) to diminish surface irregularities of the residual stromal bed after surgery with less pain, faster re-epithelialization, and better postoperative visual acuity. In this article, we report the first case of HSK following SmartSurf ^ACE^ without history of herpetic eye disease.

**Case presentation:**

A 21-year-old woman underwent bilateral SmartSurf^ACE^ without history of clinical herpetic infection, active eye disease, or systemic disease. Mild superficial punctate keratitis occurred on the tenth postoperative day. The condition was not improved by ophthalmic drugs of anti-inflammation or epithelial healings. Dendritic corneal ulcer appeared within one month, which is the commonly recognized clinical manifestation of herpes simplex keratitis. The patient was managed with topical and systemic antiviral agents. After nine days of antiviral therapy, the lesion healed up, remaining mild stromal scarring in both eyes ultimately.

**Conclusion:**

Herpes simplex keratitis is a rare but sight-threatening complication following refractive surgery. For the ocular irritation symptoms of postoperative patients, we should consider the possibility of HSK and give timely treatment.

## Background

HSK is the most prevalent infectious keratitis in the world, which is mostly caused by HSV type I. The infection can be divided into two types: primary infection and recurrent infection. 60% to 90% of people in the world carry HSV-1 antibodies. Many people have latent virus, but do not show clinical manifestation. Recurrent infection is caused by reactivation of latent virus [1].

Some concern remains regarding the potential reactivation of latent herpetic eye disease following refractive surgery. Although few cases have been documented, the incidence of postoperative keratitis following refractive surgery still occurs in 0.02–0.2% [2]. To our knowledge, HSV reactivation has not been reported in SmartSurf ^ACE^ [3]. In this article, we report a clinical picture and treatment outcomes of HSK in a patient who underwent bilateral SmartSurf ^ACE^ for the correction of myopia and astigmatism.

## Case presentation

A 21-year-old woman underwent bilateral SmartSurf ^ACE^ for correction of myopia and astigmatism. She had no history of clinical herpetic infection, active eye disease, or systemic disease. Preoperative refraction was -6.25/-0.75 × 30 in the right eye and -6.00/-1.25 × 158 in the left eye. Uncorrected visual acuity (UCVA) was 20/500, and best spectacle-corrected visual acuity (BSCVA) was 20/20 in both eyes. Slit-lamp examination was normal. Pachymetry readings were 572 mm in the both eyes. Corneal topography (Pentacam, Oculus) showed regular maps with simulated keratometry (SimK) readings of 41.16/42.19 @ 7 in the right eye and 41.22/42.43 @ 175 in the left eye.

The surgery was assisted by the Amaris 500 excimer laser. The epithelial thickness was set to 55 μm in the center and 65 μm in the periphery in the 6.5 mm diameter range. After laser ablation, a bandage contact lens was applied. The patient was treated with Levofloxacin eye drops, prednisolone acetate ophthalmic suspension, pranoprofen eye drops and 0.3% sodium hyaluronate drops.

One week after SmartSurf ^ACE^, the patient reported no discomfort. UCVA was 20/25 in both eyes. After the corneal epithelium became normal, we removed the bandage contact lens, and prescribed fluorometholone eye drops 4 times per day and 0.3% sodium hyaluronate drops every 2 h.

On the tenth postoperative day, the patient had symptoms of stinging pain and foreign body sensation. Although UCVA was still 20/25, mild superficial punctate keratitis occurred in both eyes. Deproteinized calf blood extract eye drops and eye gel were used to reconstruct corneal surface.

After three days of treatment, vision was reduced in both eyes. UCVA was decreased to 20/40. Cornea epithelial defect and superficial spotted hazes were observed in both eyes. The patient was initially treated for 1 week with fluorometholone eye drops, 0.3% sodium hyaluronate drops, deproteinized calf blood extract eye drops, deproteinized calf blood extract eye gel, and wore a bandage contact lens again.

**Fig. 1:**
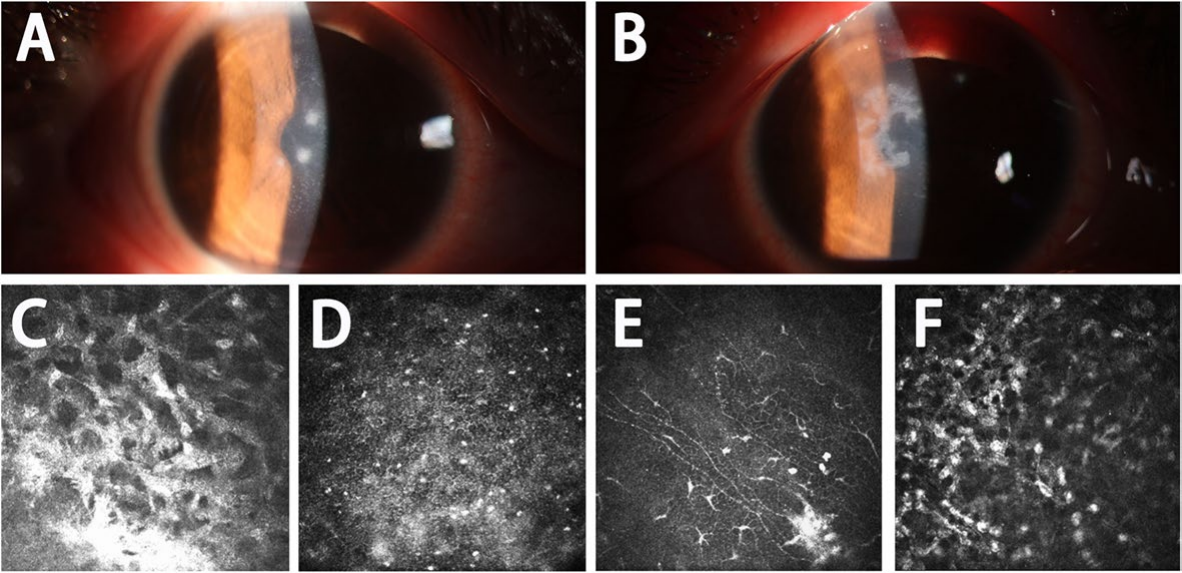
Slit-lamp examination and in vivo confocal microscopy of two eyes performed on the 24th postoperative day. **A** Three nummular sub-epithelial infiltrates were presented on the cornea in the right eye. **B** A dendritic corneal ulcer appeared in the left eye. **C**-**D** In vivo confocal microscopy showed corneal epithelial edema and anterior stromal edema in the right eyes. **E**–**F** Decreased sub basal corneal nerve density, infiltration of inflammatory cells in corneal stroma were observed in the left eye

On the 24th postoperative day, pain, photophobia, tearing, and reduced vision appeared in her eyes, which lasted 1 day. Three nummular sub-epithelial infiltrates were presented on the cornea in the right eye. A dendritic corneal ulcer appeared in the left eye (Fig. 1). Fluorescein staining was both positive. A retrospective detailed history revealed that the patient was accustomed to staying up late, lived with poor sanitation, and suffered from mental stress due to school pressures, which led to decreased immunity and catching a cold two weeks after surgery. Further, postoperative topical corticosteroid was used in both eyes. HSK was considered, and we decided on hospitalization for the patient. With removal of the contact lens and cessation of flumirone eye drops, the patient was treated with oral valaciclovir 300 mg tablets 2 times per day, ganciclovir eye drops, and 0.3% sodium hyaluronate drops every 2 h, ganciclovir eye gel 2 times per day, tacrolimus eye drops 4 times per day, and deproteinized calf blood extract eye gel once before bedtime.

In vivo confocal microscopy revealed corneal epithelial edema and anterior stromal edema in both eyes. Decreased sub basal corneal nerve density, infiltration of inflammatory cells, and Langerhans cells in the corneal stroma were observed in the left eye. No abnormal changes were observed in the corneal endothelium or amoeba infection in either eye (Fig. 1).

After two days of hospitalization, the patient complained of reduced irritation. The corneal epithelial defect had decreased in size. Because slow improvement was observed, HSK was not suspected. One week later, the dendritic corneal ulcer had become mild and the staining range of fluorescein staining was reduced.

On the ninth day of hospitalization, the patient reported no discomfort. UCVA had improved to 20/20 and 20/25. Corneal fluorescein staining was negative with complete resolution of the corneal edema. The lesion healed with only mild corneal macula remaining in the right eye. The dendritic corneal ulcer healed and replaced by a fibrotic stromal scarring the left eye. As the disease activity completely subsided, the patient was discharged from hospital and maintained on valaciclovir 300 mg tablets 2 times per day for 2 weeks; flumirone eye drops every 2 h, tapering gradually, ganciclovir eye drops and 0.3% sodium hyaluronate drops 4 times per day, and ganciclovir eye gel once before bedtime.

At the 9-month follow-up, HSK had not recurred, and only a minimal haze at the corneal interface was seen. UCVA was 20/22 and 20/33, while BSCVA was 20/20 in both eyes. The patient was advised to continue with regular review. 

## Discussion and conclusions

SmartSurf ^ACE^ is a refractive surgery used to correct myopia and astigmatism through removing the corneal epithelium in one step by SPT, which simplifies the procedure and avoids the complications of a corneal flap [4, 5].

We present the first case with HSK after SmartSurf ^ACE^. Many people have latent virus with no clinical manifestation. 60% to 90% of people worldwide carry HSV-1 antibody.1% to 6% of primary infections have clinical manifestations [1].Latent herpetic oculopathy after refractive surgery has been reported in the literatures [6–8].Two large retrospective studies revealed that the incidence rate of HSK after PRK and LASIK was much higher than that of the general population [9].

Some researches have showed that excimer laser can stimulate the reactivation of potential HSV [7].Studies have demonstrated that reactivation may appear in patients without a history of HSK [6, 8, 10].In our case, history of cold in patient, the excimer laser and the use of steroid eye drops after surgery may have acted as incentives for reactivation.

In our case presented, mild superficial punctate keratitis occurred on the tenth postoperative day. The condition was not improved by ophthalmic drugs of anti-inflammation or epithelial healings. Dendritic corneal ulcer appeared within one month, which is the commonly recognized clinical manifestation of herpes simplex keratitis. The patient was managed with topical and systemic antiviral agents. After nine days of antiviral therapy, the lesion healed up, remaining mild stromal scarring in both eyes ultimately.

We believe our case highlights the importance of patient immune status evaluation in perioperative period, and the need obtain a detailed medical history before surgery. The patients with recent bacterial or viral infections even not related to the eyes and fully resolved by the time of the surgery should be advised on potential risks of HSK after the procedure with compromised immunity.

## Data Availability

The datasets used during the current study are available from the corresponding author on reasonable request.

## Abbreviations

HSK: Herpes simplex keratitis
trans-PRK: Transepithelial photorefractive keratectomy
SPT: Smart Pulse Technology
SmartSurf^ACE^: Smart Pulse Technology assisted transepithelial photorefractive keratectomy
